# Antimicrobial activities of Ankaferd BloodStopper, hypochlorous acid, and chlorhexidine against specific organisms

**DOI:** 10.3906/sag-2107-24

**Published:** 2021-10-17

**Authors:** Ahmet KOLUMAN, Alper AKTAŞ, Selen ADİLOĞLU

**Affiliations:** 1 Department of Biomedical Engineering, Faculty of Technology, Pamukkale University, Denizli, Turkey; 2 Department of Oral Maxillofacial Surgery, Faculty of Dentistry, Hacettepe University, Ankara, Turkey

**To the Editor**,

Many antimicrobial agents can be used for the control of bacterial colonization, plaque accumulation, inflammation, and postoperative infection after the oral surgery [1]. It had been hypothesized to compare the effects of Ankaferd BloodStopper (ABS), hypochlorous acid (HOCl), and chlorhexidine gluconate (CHG) on specific microorganisms such as S*treptococcus mutans*, *Staphylococcus aureus*, *Actinomyces israelii*, and *Lactobacillus casei* to find ideal antimicrobial agent.

In this hypothesis, it was seen that higher concentration and long exposure time of all agents are more impressive at all of the microorganisms. In the long-term exposure, it was found that HOCl and CHG affect faster than ABS for *Lactobacillus casei*.

For *A. israelli*, the effect of CHG is more than ABS and HOCl (CHG > HOCl > ABS) for all organisms at short-term exposure. However, in long-term exposure, HCOl is more effective for *L. casei* and *S. aureus* than ABS but for *A. israelli* and *S. mutans*, there was not a significant difference. Although the effect of CHG on *S. mutans* started in 120 s, ABS and HOCl needed more time to affect. Coleghon et al. showed that CHG had a larger inhibition zone than ABS at both long- and short-term exposure similar to our study [2]. In the long-term, effectiveness of CHG continued for all microorganisms in this study (Table 1) (Figure).

In disc diffusion, lower concentrations of all agents were more effective for microorganisms. ABS had better results for *A. israelli* and *L. casei* than other agents. At disc diffusion, CHG was worse than ABS and HOCl (Table 2).

Barry et al. applied CHG on 1100 strains of gram-positive and gram-negative bacteria and the resistance to CHG is seen low [3]. Sensitivity of *Streptococcus mutans* to CHG is more than sensitivity of *Lactobasillus* species to CHG dealing with pH level [4]. In this study, pH was not examined but a significant difference was not seen between *L. casei* and *S. mutans* according to the concentration and duration. Only the concentration of CHG is found as important for the efficiency on *S. mutans* group.

Cinar et al. showed that ABS had a smaller inhibition zone but at long-term exposure, the results of ABS do not differ from those of the other antimicrobial agents [2]. ABS has an additional hemostatic and antiinflamation effect, beside antimicrobial effect [5]. In this study, the time needed for the effectiveness of ABS is observed to be more than CHG and HOCl. On the other hand, ABS disc diffusion form can be preferred due to its superiority to CHG and HOCl.

ABS has pleiotropic effects on blood cells, vascular endothelium, angiogenesis, and cellular proliferation that can help the tissue regeneration and wound healing [6]. In the literature, it was shown that ABS deactivates the proliferation of the cell lines and cancer cells [7]. Therefore, the combined effect (antimicrobial, hemostatic, and improving wound accelerator) makes ABS more valuable than its equivalents.

A possible advantageous use of ABS for human health was seen on *Helicobacter pylori*, an antibiotic resistant species shown in the literature [8]. If ABS is so effective on a resistant microorganism like *H. pylori* at high concentration without any damage to the living tissues, this antibacterial agent can be a good choice for precaution of the other resistant microorganisms.

Cinar et al. observed that *Lactobasillus* species have a resistance to ABS [2]. In our study, *L. casei* was resistant to ABS in short term exposure, but it was seen that in long-term exposure and at high concentration, the effect of the ABS did not differ from that of CHG and HOCl. ABS had the same effect with CHG and HOCl for *A. israelli* in long-term exposure with high concentration but there is not sufficient research in the literature about the effect of ABS on *Actinomyces* species.

HOCl is an important antimicrobial agent that is synthesized with acidification of H_2_O_2_. In this research, it was seen that HOCl is effective on *S. aureus* more than ABS, less than CHG. Also, when the concentration (200 ppm) is higher, the efficiency of HOCl is better. Ishıhara et al. showed that when applying 100 ppm HOCl, chlorine levels can reduce by the presence of organic compounds. Therefore, if concentration is higher, chlorine level and effectiveness can be higher [9].

As a result, in long-term exposure, 3 of the agents have similar antimicrobial effects and can be preferred successfully during oral surgery. Clinician should keep in mind the tissue-friendly character of HOCl, wound healing effect of ABS, and antiplaque activity of CHX while choosing the agent to prefer for the oral surgery patients.

## Figures and Tables

**Figure:**
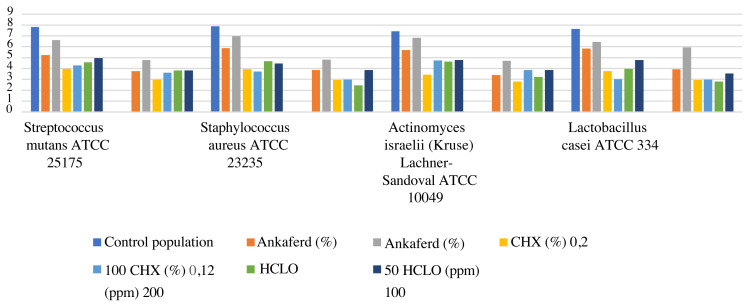
The graphic of the effects of antimicrobial agents on bacterial species.

**Table 1:** The effect of ABS, CHX, and HOCl on different bacterial species in short- and long-term.

		Control population	Ankaferd (%)		CHG (%)		HOCl (ppm)	
	Time		100	50	0.2	0.12	200	100
*Streptococcus mutans* *ATCC 25175*	5th min	7.819544	5.255273	6.60206	3.963788	4.30103	4.579784	4.944483
	10th. min		3.748188	4.78533	2.963788	3.623249	3.832509	3.812913
*Staphylococcus aureus* *ATCC 23235*	5th min	7.880814	5.892095	6.986772	3.934498	3.724276	4.662758	4.447158
	10th min		3.857332	4.826075	2.939519	2.991226	2.462398	3.863323
*Actinomyces israelii (Kruse) Lachner-Sandoval ATCC 10049*	5th min	7.431364	5.70757	6.832509	3.431364	4.732394	4.623249	4.778151
	10th min		3.39794	4.70757	2.792392	3.869232	3.230449	3.845098
*Lactobacillus casei ATCC 334*	5th min	7.653213	5.838849	6.431364	3.770852	3	3.973128	4.78533
	10th minute		3.929419	5.94939	2.944483	2.963788	2.819544	3.544068

Green = Active biocidal applications decrease at least 3 log according to CLSI and EUCAST

Yellow = Insufficient applications defined by CLSI and EUCAS.

**Table 2:** The effect of disc diffusion of ABS, CHX, and HOCl on bacterial species.

	Ankaferd (%)		CHX (%)		HCLO (ppm)	
	100	50	0.2	0.12	200	100
*Streptococcus mutans ATCC 25175*	18	13	19	15	17	14
*Staphylococcus aureus ATCC 23235*	19	11	19	14	13	11
*Actinomyces israelii (Kruse) Lachner-Sandoval ATCC 10049*	11	5	16	14	12	8
*Lactobacillus casei ATCC 334*	9	6	15	13	11	10
			Yellow = Resistant or intermittent			
			Green = Sensitive			

## References

[1] Zambon JJ Ciancio SG Mather ML Charles CH The effect of an antimicrobial mouthrinse on early healing of gingival flap surgery wounds Journal of Periodontology 1989 60 1 31 4 10.1902/jop.1989.60.1.31 2921710

[2] Cinar C Odabas ME Akca G Isik B Antibacterial effect of a new haemostatic agent on oral microorganisms Journal of Clinical and Experimental Dentistry 2012 4 3 e151 5 10.4317/jced.50750 24558546 PMC3917639

[3] Barry AL Fuchs PC Brown SD Lack of effect of antibiotic resistance on susceptibility of microorganisms to chlorhexidine gluconate or povidone iodine European Journal of Clinical Microbiology & Infectious Diseases 1999 18 12 920 1 10.1007/s100960050434 10691210

[4] Cleghorn B Bowden GH The effect of pH on the sensitivity of species of Lactobacillus to chlorhexidine and the antibiotics minocycline and spiramycin Journal of Dental Research 1989 68 7 1146 50 10.1177/00220345890680070201 2632598

[5] Gul M Gunay A Tanik A An evaluation of the effects of caffeic acid phenethyl ester and Ankaferd blood stopper on secondary wound healing of oral mucosal tissue Turkish Journal of Medical Science 2020 50 1 248 57 10.3906/sag-1908-114 PMC7080345 31769639

[6] Turk S Malkan UY Ghasemi M Hocaoglu H Mutlu D Growth inhibitory activity of Ankaferd hemostat on primary melanoma cells and cell lines SAGE Open Medicine 2017 5 2050312116689519 10.1177/2050312116689519 28293423 PMC5336193

[7] Ghasemi M Okay M Malkan UY Turk S Jabbar J Ankaferd Hemostat Affects Etoposide Resistance of the Malignant Melanoma Cells International Journal of Hematology and Oncology 2020 30 1 43 53 10.4999/uhod.203965

[8] Ciftciler R Koluman A Haznedaroglu IC Akar N Effects of Ankaferd Hemostat on Helicobacter pylori strains and antibiotic resistance Turkish Journal of Medical Science 2019 49 1 347 55 10.3906/sag-1807-206 PMC7350798 30761849

[9] Ishihara M Murakami K Fukuda K Nakamura S Kuwabara M Stability of Weakly Acidic Hypochlorous Acid Solution with Microbicidal Activity Biocontrol Science 2017 22 4 223 7 10.4265/bio.22.223 29279579

